# Transition from Direct-View to Totally Endoscopic Mitral Valve Surgery in an Experienced Minimally Invasive Center: A Propensity-Matched Analysis

**DOI:** 10.3390/life16071142

**Published:** 2026-07-09

**Authors:** Andrzej Klapkowski, Aleksandra Stańska, Igor Tomczyk, Radosław Targoński, Wojciech Karolak

**Affiliations:** 1 Department of Cardiac Surgery, Faculty of Medicine, Medical University of Gdansk, ul. Skłodowskiej-Curie 3A, 80-210 Gdańsk, Poland; 2 Division of Quality of Life Research, Department of Psychology, Faculty of Health Sciences, Medical University of Gdansk, Ul. Skłodowskiej-Curie 3A, 80-210 Gdańsk, Poland; 3 First Department of Cardiology, Faculty of Medicine, Medical University of Gdansk, ul. Skłodowskiej-Curie 3A, 80-210 Gdańsk, Poland

**Keywords:** mitral valve surgery, endoscopic cardiac surgery, minimally invasive cardiac surgery, mitral valve repair, propensity score matching, cardiopulmonary bypass

## Abstract

**Background:** Totally endoscopic mitral valve surgery has gained increasing interest as an evolution of minimally invasive cardiac surgery. However, concerns remain regarding the implementation of endoscopic techniques and their potential impact on procedural safety and operative efficiency. The present study aimed to evaluate the early experience of transitioning from direct-view minimally invasive mitral surgery to a totally endoscopic approach in an experienced minimally invasive center. **Methods:** A retrospective analysis of consecutive patients undergoing minimally invasive mitral valve surgery was performed. The study included 209 patients, comprising 36 totally endoscopic and 173 direct-view minimally invasive procedures. Propensity score matching was performed using age, left ventricular ejection fraction, and New York Heart Association functional class, resulting in 36 matched pairs. Continuous variables were compared using Student’s *t*-test and categorical variables using Fisher’s exact test. **Results:** After propensity score matching, baseline characteristics were well balanced between groups. The endoscopic cohort demonstrated significantly shorter cardiopulmonary bypass time compared with the direct-view group (120.4 ± 44.3 vs. 153.1 ± 40.1 min; *p* = 0.001). Aortic cross-clamp time was also significantly shorter in the endoscopic cohort (77.1 ± 23.9 vs. 97.8 ± 32.0 min; *p* = 0.002). Postoperative outcomes remained comparable between groups. The incidence of de novo atrial fibrillation was similar (11.1% vs. 13.9%; *p* = 1.0), as were blood transfusion requirements (47.2% vs. 50.0%; *p* = 1.0). Major postoperative complications occurred infrequently in both cohorts. Procedural success was achieved in 94.4% of direct-view procedures and 100% of endoscopic procedures (*p* = 0.493). **Conclusions:** Transition from direct-view minimally invasive mitral surgery to a totally endoscopic approach was associated with significantly shorter cardiopulmonary bypass and aortic cross-clamp times while maintaining comparable early postoperative outcomes. These findings suggest that implementation of totally endoscopic mitral surgery can be achieved safely in experienced minimally invasive centers and may be associated with shorter operative times while maintaining comparable early postoperative outcomes. Further multicenter studies are warranted to better define the impact of endoscopic techniques on operative performance and clinical outcomes.

## 1. Introduction

Mitral valve repair remains the gold standard for the treatment of degenerative mitral regurgitation due to its favorable long-term outcomes and preservation of native valve function [1,2,3]. Nevertheless, mitral valve replacement continues to play an important role in patients with severely degenerated or stenotic valves, failed previous repair, or particularly complex valve pathology.

Over the past two decades, minimally invasive mitral valve surgery has been increasingly adopted worldwide, demonstrating safety, feasibility, and favorable postoperative outcomes in appropriately selected patients. In many experienced centers, right minithoracotomy has become a standard approach for mitral and tricuspid valve procedures, offering reduced surgical trauma while maintaining excellent operative exposure [4,5].

In our institution, minimally invasive mitral and tricuspid valve surgery through direct-view minithoracotomy has been routinely performed for nearly 10 years and remains the preferred approach in patients suitable for peripheral cannulation and without severe chest wall deformities [6]. In March 2025, despite excellent outcomes achieved with direct-view surgery, our team implemented a totally endoscopic 3D-assisted approach.

The totally endoscopic technique offers several potential advantages, including enhanced visualization of the operative field, improved control of port placement, avoidance of rib retractors, and potentially superior surgeon ergonomics. However, critics of the endoscopic approach emphasize the substantial learning curve associated with endoscopic surgery, particularly the need for advanced screen-hand coordination and dedicated equipment [7,8,9].

Importantly, our transition to a totally endoscopic technique occurred within a highly experienced minimally invasive mitral surgery program. We hypothesized that extensive prior experience with direct-view minithoracotomy could facilitate a safe implementation of the endoscopic approach without compromising perioperative outcomes—and thus is the main premise of the present manuscript.

Therefore, the present study aimed to provide a real-world assessment of the early institutional experience associated with the transition from direct-view minimally invasive mitral valve surgery to a totally endoscopic approach in an experienced surgical team.

## 2. Methods

### 2.1. Patient Selection and Data Collection

The study included consecutive patients undergoing minimally invasive mitral valve surgery at our institution, including both totally endoscopic and direct-view minimally invasive procedures. Clinical, operative, and echocardiographic data were retrospectively collected from institutional databases and medical records.

### 2.2. Surgical Technique

Minimally invasive mitral valve surgery was performed under general anesthesia using either single- or double-lumen endotracheal intubation according to surgeon preference. Patients were positioned in a 30-degree right semi-supine position. Preoperative computed tomography was routinely performed in all patients to assess chest anatomy and suitability for peripheral cannulation.

Cardiopulmonary bypass was established via femoro-femoral cannulation. In patients with significant peripheral arterial disease, heavily calcified vessels, or unsuitable femoral anatomy, direct axillary artery cannulation was utilized.

Surgical access was achieved through a right minithoracotomy in all cases. In the totally endoscopic group, an additional lateral port within the same intercostal space was created for insertion of a three-dimensional endoscopic camera. In this cohort, rib spreading was completely avoided, and the entire procedure was performed under endoscopic visualization. Direct-view minimally invasive procedures were performed following the introduction of the endoscopic program. Endoscope use was determined by its availability. No other selection criteria were applied.

The ascending aorta was cross-clamped using a transthoracic Chitwood clamp. Myocardial protection was achieved using either HTK Bretschneider or del Nido cardioplegia solution according to surgeon preference.

Mitral valve repair was performed using standard techniques, including PTFE neochord implantation, indentation closure, comissuroplasty and annuloplasty ring implantation. Whenever repair was not feasible or indicated, mitral valve replacement was performed using conventional techniques. Concomitant procedures, including tricuspid valve repair, patent foramen ovale closure, and left atrial appendage management, were performed when clinically indicated.

### 2.3. Definitions

Operative safety was defined as the absence of major perioperative complications, including mortality, stroke, reoperation for bleeding, and conversion to sternotomy.

Procedural success was defined as successful completion of the planned minimally invasive procedure with satisfactory early echocardiographic result. In patients undergoing mitral valve repair, procedural success additionally required no greater than mild residual mitral regurgitation on postoperative echocardiography [10]. In patients scheduled for mitral valve replacement, procedural success required successful valve implantation without paravalvular leak.

### 2.4. Statistical Analysis

To reduce baseline differences between patients undergoing direct-view and endoscopic minimally invasive mitral valve surgery, propensity score matching was performed. The propensity score was estimated using a logistic regression model including age, left ventricular ejection fraction, and New York Heart Association functional class.

Because of the relatively small number of patients undergoing totally endoscopic surgery, the propensity score model was intentionally restricted to a limited number of clinically relevant baseline variables in order to reduce the risk of model overfitting and instability. Age, left ventricular ejection fraction, and NYHA functional class were selected as key indicators of functional status and overall operative risk.

One-to-one nearest-neighbor matching without replacement was applied using a caliper width of 0.2. Covariate balance after matching was assessed using standardized mean differences.

Continuous variables were compared using Student’s *t*-test and categorical variables using Fisher’s exact test. A *p*-value < 0.05 was considered statistically significant. All statistical analyses were performed using R software (version 4.5.3).

## 3. Results

### 3.1. Study Population

A total of 209 patients were included in the analysis, comprising 36 endoscopic procedures and 173 direct-view minimally invasive procedures. Following propensity score matching, 36 well-balanced pairs were obtained and included in the final analysis.

### 3.2. Baseline Characteristics After Matching

**Figure 1:**
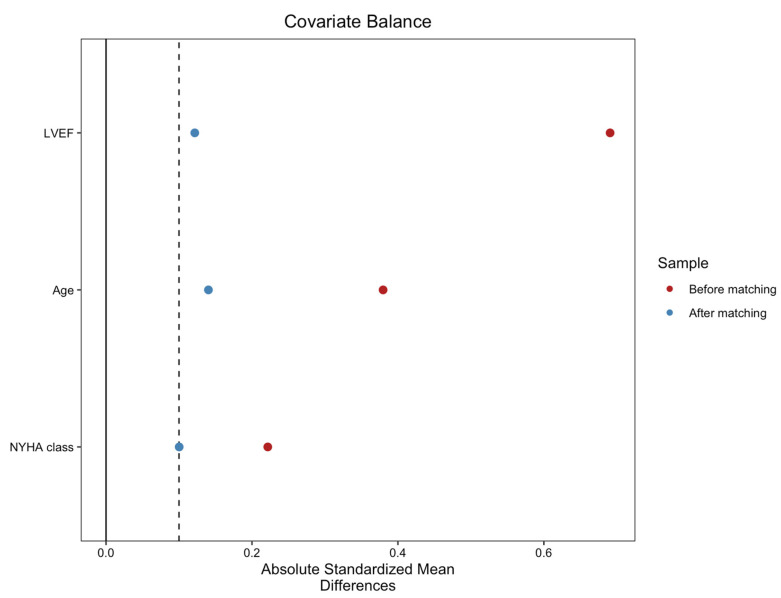
Standardized mean differences before and after propensity score matching. The dashed vertical line represents the threshold of an absolute standardized mean difference of 0.1, indicating acceptable covariate balance.

After matching, baseline demographic and clinical characteristics were comparable between groups (Table 1). Propensity score matching substantially reduced baseline imbalances between groups, resulting in acceptable covariate balance across the matching variables (Figure 1).

Mean age was 52.7 ± 14.7 years in the direct-view group and 55.0 ± 16.8 years in the endoscopic group. Left ventricular ejection fraction remained preserved and similar between cohorts (57.0 ± 7.0% vs. 57.8 ± 7.1%, respectively), while serum creatinine concentration did not differ substantially between groups.

The distribution of sex was balanced following matching, with female patients accounting for 44.4% and 50.0% of the direct-view and endoscopic cohorts, respectively. Valve repair represented the predominant surgical strategy in both groups, accounting for 80.6% and 86.1% of procedures, respectively.

Valve morphology was overall comparable between cohorts. Barlow disease and fibroelastic deficiency represented the most common etiologies in both groups. Ring size was slightly larger in the endoscopic cohort, although this difference did not reach statistical significance.

### 3.3. Operative and Postoperative Outcomes

The endoscopic approach was associated with significantly shorter cardiopulmonary bypass time compared with the direct-view approach (120.4 ± 44.3 vs. 153.1 ± 40.1 min, *p* = 0.001). Aortic cross-clamp time was also significantly shorter in the endoscopic cohort (77.1 ± 23.9 vs. 97.8 ± 32.0 min, *p* = 0.002), corresponding to a mean reduction of approximately 20 min compared with the direct-view group.

Postoperative outcomes were comparable between groups (Table 2). The incidence of de novo atrial fibrillation was low and similar between cohorts (11.1% vs. 13.9%, *p* = 1.0). Likewise, no significant differences were observed regarding transfusion requirements or major postoperative complications.

Major postoperative complications were uncommon in both groups. In the matched cohort, stroke did not occur in either group. Pacemaker implantation and in-hospital mortality occurred in one patient each in the direct-view group and in no patients in the endoscopic group. Conversion occurred in one patient in each group, while rethoracotomy was observed in three direct-view patients and four endoscopic patients. The composite major complication rate was numerically lower in the endoscopic group, although this difference was not statistically significant (11.1% vs. 16.7%; *p* = 0.735). Operative safety, defined as absence of mortality, stroke, conversion, and rethoracotomy, was comparable between groups (88.9% vs. 86.1%; *p* = 1.00).

Procedural success was achieved in 34 patients (94.4%) in the direct-view group and in all patients (100.0%) in the endoscopic group, with no statistically significant difference between groups (*p* = 0.493).

## 4. Discussion

In the present study, the endoscopic approach to mitral valve surgery was associated with significantly shorter cardiopulmonary bypass and aortic cross-clamp times compared with the direct-view minimally invasive approach. Importantly, these improvements in operative efficiency were not accompanied by an increase in postoperative complications, transfusion requirements, or other adverse early postoperative outcomes. Taken together, these findings suggest that the transition from direct-view minimally invasive surgery to a totally endoscopic approach can be performed safely in an experienced center while potentially improving procedural efficiency. We believe that extensive prior experience with direct-view minimally invasive surgery was an important factor facilitating safe implementation of the totally endoscopic approach.

The reduction in cardiopulmonary bypass time represents one of the most clinically relevant findings of the present analysis. Prolonged extracorporeal circulation has been associated with systemic inflammatory response, coagulopathy, organ dysfunction, and adverse postoperative outcomes [11]. Consequently, reductions in bypass duration may have meaningful implications not only for operative workflow but also for perioperative recovery. In the present matched cohort, the endoscopic approach reduced cardiopulmonary bypass time by more than 30 min while maintaining comparable early safety outcomes.

Similarly, the endoscopic cohort demonstrated significantly shorter aortic cross-clamp times, with a reduction exceeding 20 min compared with the direct-view group. Prolonged myocardial ischemic time has been associated with impaired postoperative myocardial recovery and perioperative morbidity. Therefore, shorter ischemic intervals may contribute to improved myocardial protection and procedural efficiency. The consistency of reductions observed in both cardiopulmonary bypass and cross-clamp times strengthens the overall validity of the findings and suggests that the observed differences likely reflect genuine procedural advantages rather than random variation.

Importantly, these findings contrast with earlier reports describing prolonged operative times during the initial adoption of endoscopic mitral valve surgery. Historically, totally endoscopic approaches have been associated with substantial learning curves related to port-based access, endoscopic visualization, and instrument coordination. However, more recent reports indicate that operative times decrease significantly with growing institutional experience and procedural standardization.

The present observations should therefore be interpreted in the context of the substantial institutional experience with minimally invasive mitral surgery within the study center. Endoscopic procedures were introduced in a team already highly experienced in direct-view minimally invasive mitral operations. As such, implementation of the endoscopic technique represented a continuation and refinement of an established minimally invasive program rather than the beginning of minimally invasive experience itself. This likely mitigated the impact of the learning curve traditionally associated with totally endoscopic surgery and allowed the technical advantages of enhanced endoscopic visualization to translate into measurable procedural efficiency.

Nevertheless, the observed reductions in cardiopulmonary bypass and cross-clamp times should be interpreted cautiously. The present study was not designed to establish superiority of one approach over another, and the observational design, limited sample size, and potential case-selection effects may have influenced the findings. Therefore, the results should be viewed primarily as evidence that implementation of a totally endoscopic approach within an experienced minimally invasive program can be achieved without an apparent early safety penalty, rather than definitive proof of improved procedural performance.

Several procedural aspects may additionally contribute to improved operative performance during endoscopic surgery. Enhanced visualization provided by the endoscopic camera allows detailed inspection of port and thoracotomy sites throughout the procedure and may facilitate more efficient hemostasis. In direct-view minimally invasive procedures, hemostatic control is often performed during cardiopulmonary bypass under conditions of limited exposure and restricted lung ventilation. Improved visualization in endoscopic surgery may therefore simplify this stage of the procedure and potentially contribute to shorter bypass and cross-clamp times.

Importantly, the observed procedural efficiency was not associated with an increased risk of adverse outcomes. Procedural success rates were also high in both groups, exceeding 94%, with no significant between-group differences. The incidence of postoperative atrial fibrillation, transfusion requirements, and other major postoperative complications remained comparable between groups, supporting the early safety of the endoscopic approach in experienced hands [12,13,14]. These findings are consistent with the broader literature suggesting that minimally invasive and endoscopic mitral valve surgery can be performed safely and reproducibly in specialized centers [13,15,16,17,18].

## 5. Study Limitations

The present study has several limitations that should be acknowledged. First, despite the use of propensity score matching, the analysis remains observational in nature and residual confounding cannot be entirely excluded. Patient allocation to the endoscopic or direct-view approach was not randomized and may have been influenced by anatomical, technical, or surgeon-related considerations. Therefore, selection bias cannot be excluded.

Second, the endoscopic procedures were introduced later than the majority of direct-view procedures. Consequently, improvements in operative times may partially reflect temporal factors, increasing institutional experience, workflow optimization, and accumulated team expertise rather than the surgical approach alone.

Third, the relatively small number of matched pairs limited statistical power, particularly for infrequent postoperative complications, and restricted the number of covariates that could be included in the propensity score model.

Finally, the study reflects the experience of a single high-volume center with extensive expertise in minimally invasive mitral surgery, which may limit generalizability to centers at earlier stages of endoscopic program development.

## 6. Conclusions

In conclusion, the transition from direct-view minimally invasive mitral surgery to a totally endoscopic approach was associated with significantly shorter cardiopulmonary bypass and aortic cross-clamp times while maintaining comparable early postoperative safety outcomes. These findings suggest that, in experienced minimally invasive centers, implementation of endoscopic mitral surgery can be achieved safely and may improve procedural efficiency. Further studies involving larger multicenter cohorts are warranted to better define the impact of endoscopic techniques on operative performance and clinical outcomes.

## Figures and Tables

**Table 1:** Baseline and valve characteristics after propensity score matching.

Variable	Direct-View (*n* = 36)	Endoscopic (*n* = 36)	*p*-Value
Age, years	52.7 ± 14.7	55.0 ± 16.8	0.52
Female sex, *n* (%)	16 (44.4)	18 (50.0)	0.81
Left ventricular ejection fraction, %	57.0 ± 7.0	57.8 ± 7.1	0.80
Creatinine, mg/dL	0.89 ± 0.24	0.93 ± 0.23	0.47
NYHA class	2.69 ± 0.47	2.75 ± 0.44	0.64
Valve repair, *n* (%)	29 (80.6)	31 (86.1)	0.75
Valve replacement, *n* (%)	7 (19.4)	5 (13.9)	0.75
Ring size	33.5 ± 4.0	35.0 ± 4.9	0.17
Tricuspid valve repair, *n* (%)	9 (25.0)	8 (22.2)	1.00
PFO closure, *n* (%)	7 (19.4)	8 (22.2)	1.00
*Valve morphology*			
Barlow disease, *n* (%)	12 (33.3)	16 (44.4)	0.775 *
Fibroelastic deficiency (FED), *n* (%)	7 (19.4)	4 (11.1)	
FED+, *n* (%)	10 (27.8)	7 (19.4)	
Functional mitral regurgitation, *n* (%)	4 (11.1)	6 (16.7)	
Mitral stenosis, *n* (%)	1 (2.8)	2 (5.6)	
Other/not specified, *n* (%)	2 (5.6)	1 (2.8)	

* *p*-value calculated for overall valve morphology distribution using Fisher’s exact test.

**Table 2:** Operative and postoperative outcomes after propensity score matching.

Variable	Direct-View (*n* = 36)	Endoscopic (*n* = 36)	*p*-Value
Cardiopulmonary bypass time, min	153.1 ± 40.1	120.4 ± 44.3	0.001
Aortic cross-clamp time, min	97.8 ± 32.0	77.1 ± 23.9	0.002
De novo atrial fibrillation, *n* (%)	5 (13.9)	4 (11.1)	1.00
Blood transfusion, *n* (%)	18 (50.0)	17 (47.2)	1.00
Stroke, *n* (%)	0 (0.0)	0 (0.0)	Not tested
Pacemaker implantation, *n* (%)	1 (2.8)	0 (0.0)	1.00
Rethoracotomy, *n* (%)	3 (8.3)	4 (11.1)	1.00
Conversion, *n* (%)	1 (2.8)	1 (2.8)	1.00
In-hospital mortality, *n* (%)	1 (2.8)	0 (0.0)	1.00
Composite major complications, *n* (%)	6 (16.7)	4 (11.1)	0.735
Operative safety, *n* (%)	31 (86.1)	32 (88.9)	1.00
Procedural success, *n* (%)	34 (94.4%)	36 (100%)	0.493

## Data Availability

The data presented in this study are available on request from the corresponding author. The data are not publicly available due to privacy and ethical restrictions related to clinical patient records.
